# A Troubled Past? Reassessing Ethics in the History of Tissue Culture

**DOI:** 10.1007/s10728-015-0304-0

**Published:** 2015-08-04

**Authors:** Duncan Wilson

**Affiliations:** Centre for the History of Science, Technology and Medicine (CHSTM), University of Manchester, The Simon Building, Brunswick Street, Manchester, M13 9PL UK

**Keywords:** Ethics, HeLa, History, Informed consent, Tissue culture

## Abstract

Recent books, articles and plays about the ‘immortal’ HeLa cell line have prompted renewed interest in the history of tissue culture methods that were first employed in 1907 and became common experimental tools during the twentieth century. Many of these sources claim tissue cultures like HeLa had a “troubled past” because medical researchers did not seek informed consent before using tissues in research, contravening a long held desire for self-determination on the part of patients and the public. In this article, I argue these claims are unfair and misleading. No professional guidelines required informed consent for tissue culture during the early and mid twentieth century, and popular sources expressed no concern at the widespread use of human tissues in research. When calls for informed consent did emerge in the 1970s and 1980s, moreover, they reflected specific political changes and often emanated from medical researchers themselves. I conclude by arguing that more balanced histories of tissue culture can make a decisive contribution to public debates today: by refuting a false dichotomy between science and its publics, and showing how ethical concepts such as informed consent arise from a historically specific engagement between professional and social groups.

## Introduction: “Tissues with a Troubled Past”?

In February 1951, Henrietta Lacks, a young African-American woman, entered the gynaecology clinic at Johns Hopkins Hospital in Baltimore to seek treatment for a recently discovered cervical tumour. She signed a consent form allowing doctors to undertake her treatment and, after 2 days in observation, had surgery to fit a radium tube [24]. Before he applied the radium tube to Lacks’s cervix, the attending surgeon took a small biopsy and placed it in a glass dish. A resident then took this carcinoma sample to George Gey, a medical researcher who had shared a series of makeshift laboratories at Johns Hopkins with his wife Margaret since the early 1930s.

Like many researchers in the United States and beyond, George Gey utilised contacts with local surgeons to obtain samples for the technique known as ‘tissue culture’, which he believed provided an ideal tool for research into cancer. Tissue culture involves the growth of human or animal tissues and cells in laboratory conditions, or ‘in vitro’. It was first employed by the Yale embryologist Ross Harrison in 1907, who cultured frog embryo cells in a lymph suspension to settle a dispute into nerve growth, and was then taken up and popularised by the Nobel Prize winning biologist Alexis Carrel, who worked at the Rockefeller Institute for Medical Research in New York. Carrel and his assistant Montrose Burrows argued that tissue culture was a powerful medical tool and established thousands of cultures with healthy and diseased tissues taken from experimental animals and, notably, from patients who had undergone operations at nearby hospitals. They argued that human cultures, in particular, offered a valuable “new method for the study of human cancers” since they gave findings that were directly applicable to patients and allowed scientists to “study the growth of various tumours and to follow all morphological characters and changes of the cancerous and other cells during life” [12].

These bold claims were undermined, however, by the fact that human tissues rarely survived more than a few days in vitro. Scientists argued this was due to infection or problems with nutrient media, and maintained that once they solved these problems “then it is reasonable to assume that human tissue can be cultivated in vitro for an indefinite period” [29: 602]. They drew encouragement from an seemingly immortal strain of chick heart cells that Carrel had cultured in 1912, which continued to divide in Rockefeller laboratories for over 30 years and received regular ‘birthday’ notices in newspapers.^1^ George Gey, for one, believed human tissue simply needed a better in vitro environment in order to grow indefinitely. From the early 1940s, he added antibiotics to the culture medium to prevent infection, and began to explant material in a complicated ‘roller tube’ system that constantly rotated tissues, regulated carbon dioxide levels and kept pH at a healthy level. But none of the tissues that Gey added to his new system survived long, and the prospect of indefinitely culturing human material remained a distant one. When the material from Henrietta Lacks arrived in his lab, it was cultured more out of routine than expectation.

But for reasons that remain unclear, the sample that Gey codenamed ‘HeLa’ thrived in vitro and became the first immortal ‘cell line’ derived from humans. Cell lines such as HeLa offered a homogenous, self-replicating and simplified experimental system, which could be easily transported and ensured researchers were less reliant on the “good offices” of surgeons for their materials [20]. Gey was soon inundated with requests for HeLa and became well-known for travelling widely and freely distributing it to colleagues. By the mid 1950s, thanks largely to Gey’s efforts, HeLa had been incorporated into many national and international research projects, including cancer research and assays for Jonas Salk’s new polio vaccine, and the journal *Cancer* noted it had quickly become a “widely used” biomedical tool [18].

Thanks to its use in high profile and important research, HeLa quickly acquired what Hannah Landecker [27: 161] calls a “remarkable public life”. It made its public debut on 4 October 1951, when Gey held up a vial of the cell line on national television and hailed it as a powerful new weapon in the fight against disease. This short programme was one of the few instances when HeLa was not publicly linked to, or conflated with, the patient who had provided the cells, who died of cancer at Johns Hopkins the same night as Gey appeared on television. Newspapers often asked Gey for information on HeLa’s origins, to give their stories an important “human interest element” [27: 162], although their efforts were always rebuffed. This was a time of widespread public confidence in medicine, thanks to the development of antibiotics and vaccines for diseases such as polio, and journalists nevertheless went ahead and wrote positive reports that presented HeLa as the source of untold ‘magic bullets’. Amidst this celebratory backdrop, they regularly memorialised ‘Henrietta Lakes’, ‘Helen L.’ or ‘Helen Larson’ as an unsung heroine whose untimely death had helped saved thousands of lives.^2^

After this brief flurry of interest, most accounts of HeLa were confined to scientific literature from the late 1950s. It was here, in a 1971 obituary for George Gey, that Johns Hopkins doctors publicly named Henrietta Lacks as the source of HeLa: claiming her biopsy had granted her a kind of “immortality” and wondering whether “she will live forever if nurtured by the hands of future workers” [24: 947]. Press interest re-emerged later in the 1970s once scientists discovered HeLa had contaminated many other cell lines and invalidated research worth millions of dollars, including Richard Nixon’s ‘War on Cancer’. Journalists now directly mentioned Lacks and dwelt on her ethnicity, thanks largely to the fact that scientists used an enzyme marker predominantly found in African-Americans to detect HeLa contamination. Debates on contamination occurred at a time of racial unrest and civil rights campaigns, and magazines such as *Rolling Stone* regularly depicted HeLa and their source as “black and female” [27: 171].

Popular accounts of HeLa waned after the contamination issue was resolved, although the cell line was the subject of a 1996 documentary by the British filmmaker Adam Curtis and made a brief appearance in Will Self’s 2000 book *How the Dead Live*, as a talking wallpaper for houses in purgatory. This lull was temporary, however, and HeLa again became the subject of public interest following the 2010 publication of *The Immortal Life of Henrietta Lacks*, which was written by the American science journalist Rebecca Skloot. The book focussed on Henrietta Lacks’s life as a poor Baltimore housewife and explored how her family coped after learning about HeLa in the mid 1970s. It brought the story to a new audience and quickly became a best-seller across the United States and Europe, winning several non-fiction prizes and inspiring two plays that toured Britain in early 2014.

In addition to fostering renewed discussion of HeLa in newspapers and magazines, in theatres and on television, Skloot’s book also drew attention to the ethics of tissue culture, and particularly questions about whether or not scientists should obtain informed consent from patients before using their tissues in research. This had been a topical issue in bioethics since the 1980s, when John Moore sued scientists at the University of California, Los Angeles, for converting part of his spleen into a cell line without his knowledge or consent; and popular accounts of HeLa that followed this lawsuit sometimes noted that scientists “never asked” [42: 465] Lacks if she consented to the use of her biopsy in research. But Skloot ensured that questions about informed consent, or the lack of it, were now at the forefront of HeLa’s ‘public life’. The book’s dust jacket noted that Lacks’s cancer cells were “taken without her knowledge”, for instance, and Skloot reiterated that no-one “asked if she wanted to be a donor” [45: 33].

These claims now permeate the public appraisal of HeLa and its history. Reviews of Skloot’s book regularly noted that “no-one asked permission” before establishing the cell line, while publicity for Audura Onashie’s one woman play, titled *HeLa*, claimed that “without her consent or knowledge, cancerous cells were taken from Lacks’s cervix and were used to create the first ‘immortal’ human cell line” [40, 213: 459]. Some accounts simply portrayed Lacks as an “unwitting donor” [22], but others went further and openly criticised researchers for not obtaining informed consent before they established HeLa. One review of Onashile’s play, for instance, claimed the lack of consent meant that HeLa’s establishment was an “injustice” and demonstrates how medical researchers viewed Lacks “as a commodity rather than a human being” [30]. The bioethicist Arthur Caplan [10], meanwhile, framed it as a “long-standing wrong” and argued that scientists should always obtain patient consent before establishing tissue cultures to ensure that “ethics caught up to a particularly fast-moving area of science”.

These claims underpin a now widespread assumption about the HeLa story: that Lacks and her family have been mistreated by researchers, dating back to when the cell line was first established in 1951. Criticism is not only applied to HeLa, moreover, but also to the thousands of other human tissue cultures that originated from samples taken during the early and mid twentieth century. A recent *Nature* editorial pointed out that scientists routinely established tissue cultures without patient consent, even after medical associations and governments published guidelines for research involving human subjects in the 1960s and 1970s. It argued these were “tissues with a troubled past”, and claimed the only way to guarantee public trust and accountability was to require that researchers obtain “written consent for the use of leftover tissue from surgeries and other procedures—even those that have been stripped of identifiers” [8: 407].

This criticism of research practices, along with demands for measures that give patients greater control over tissues, characterises much popular and academic writing on the scientific use of human materials today. In this growing body of work, of which *The Immortal Life of Henrietta Lacks* is a prominent example, biomedical science is regularly depicted as operating against the norms and values of patients and the public. Many of these books and articles are written as ‘exposés’ of scientific malpractice, adopting language and imagery that presents human tissue research as exploitative and unethical [16]. Rebecca Skloot [45: 317], for one, closed her book by noting that the non-consented establishment of tissue cultures is troubling because “people often have a strong sense of ownership when it comes to their bodies. Even tiny scraps of them”. Writers in this ‘social unease’ genre regularly use historical examples to argue that scientists have long violated these widely held values of self-determination, justice and freedom from exploitation, and claim there is a “fearful symmetry” between the past and present, both for research practices and public responses [41]. The examples are many, and include the ‘body snatching’ scandals of the nineteenth centuries, or retention of body parts from indigenous peoples in the United States and elsewhere; [2] but the widespread criticism of HeLa and its history indicates that many writers now portray the non-consented establishment of tissue cultures as emblematic of the ways in which biomedical science seemingly rides, and has long rode, roughshod over cultural values.

But just how fair are these assumptions, and how accurate is this reading of the past? In the rest of this article, I will critique these widespread claims about the history of tissue culture. I firstly show how it is unfair to claim that the non-consented use of human tissues was an injustice that offers a “sad commentary” [44] on research ethics for much of the twentieth century. Using a variety of professional and popular sources, I argue that human tissues removed during clinical procedures were readily obtained and used in research, without any thought for patient consent or ownership, because these practices were widely seen as unproblematic. Professional manuals and research guidelines said nothing about obtaining informed consent in this period, while newspapers voiced no unease at how in vitro tissues ended up “outside of the body of which they were once part” [4].

I then detail why informed consent became increasingly central to discussion of tissue research in the late 1970s and 1980s, and show how its emergence undermines central claims of this ‘social unease’ literature. Demands for informed consent arose for historically specific reasons, I argue, and often emanated from scientists themselves. What is more, when attempts were made to survey public opinion, the responses were ambivalent and undermine claims that “individuals have genuine concerns regarding how their tissues will be used” [37]. I conclude by outlining how empirical studies on public attitudes, including more balanced histories, might benefit present debates on the biomedical uses of human tissue.

## Obtaining Human Tissues for Research *c*. 1910–1970

During the early twentieth century, the growth of fields such as embryology and histology, and the emphasis on experimentation over analysis, led biological scientists to establish greater links with animal breeders, slaughterhouses and local surgeons in order to acquire human and animal materials for their research [13]. This was certainly the case with tissue culture, which scientists across a range of disciplines viewed as a “new power” [47: 8] that would shed light on development and disease. Despite technical problems and the fact it fared less well in vitro than material taken from animals, many researchers preferred to use human tissue because they believed the results of their work “would be of more value if clearly applicable to human beings” [26: 683]. They relied on nearby surgeons for material, who either sent them tissues or allowed them to wait in surgery during an operation (scientists preferred to use tissues obtained from clinical procedures, as they generally survived longer in vitro than post-mortem material). The importance of these informal and professional contacts was highlighted in 1951 by the American biologist Margaret Murray [33: 211], who claimed in a chapter for a tissue culture manual that the “uses and advantages of cultured human tissues are many” and urged researchers to visit their local hospital to procure this “almost untapped” resource.

Murray recommended that scientists should take care not to aggravate or overburden the surgeons who provided tissues, lest they become uncooperative and withhold samples in future. Notably, however, she said nothing about obtaining consent from patients or their families. As scientists who worked in this period attest, patients had already consented to the clinical procedure and no-one thought that additional consent for research on any excised tissues was necessary [19]. This meant that scientists could simply walk into their local hospital and collect tissues provided they had permission from the resident surgeon. As part of this informal collection network, they acquired material from patients irrespective of their ethnicity or social background, as well as from themselves or their lab assistants when no operations were scheduled.

This informal network persisted despite the growing regulation of clinical procedures involving human materials from the 1950s onwards. Doctors in Britain and the United States encouraged the passage of new Acts to regulate the expanding uses of post-mortem tissues in transplantation and grafting procedures in this period. But these new laws simply stated that doctors should seek the consent of families before removing tissues from dead bodies, and said nothing about obtaining tissues from living patients during clinical procedures [51]. This was also the case with guidelines that were adopted following criticism of human experiments in the 1960s, such as the World Medical Association’s Declaration of Helsinki. These research guidelines stated that researchers should obtain informed consent before experimenting on individuals, but again overlooked research on tissues obtained during operations or biopsies. And the professional ‘whistleblowers’ who criticised research on both sides of the Atlantic in the 1960s were similarly indifferent. The American doctor Henry Beecher [9] and his British counterpart Maurice Pappworth [36] both raised questions about research involving tissues and cells, but this centred on tissue grafting experiments and the injection of cancer cells into prison inmates without full disclosure of risks. Neither said anything about the widespread collection, without consent, of tissue samples from patients who had already consented to clinical procedures.

While scientists who established tissue cultures without consent were not contravening any guidelines or professional norms, we cannot yet be certain that the practice was unproblematic. There are plenty of historical examples where medical procedures were not prohibited in guidelines, or were endorsed by specific laws, but nevertheless ran counter to the interests and values of large numbers of people. Compulsory smallpox vaccination in the nineteenth century is one [17], as is the forced sterilisation of those deemed mentally ‘unfit’ in countries such as the United States, Germany and Sweden during the early twentieth century [25]. Considering only what professional groups considered to be ethical, and failing to scrutinise whether their actions outraged contemporaries, amounts to an incomplete picture and deprives us of a standpoint from which we can accurately determine the morality of historical conduct [49]. We might be justified in saying tissue cultures had a “troubled past”, then, if there was any evidence of public unease at their establishment during the early or mid twentieth century.

But the fact remains that no-one appears to have expressed concern at the widespread collection and use of human tissues in this period. This lack of criticism did not result, as some claim, from the fact that research was hidden from the public [2]. Scientists who were keen to promote their work often wrote newspaper columns and appeared on radio or television to document how tissues “obtained from any animal or human being” [3] were being used to understand development and fight disease. These claims passed without criticism, and journalists instead wrote positive reports outlining how human tissue cultures were being used to develop vaccines and other ‘magic bullets’. In 1959, for example, *The Times* reported that British research on the harmful effects of smoking involved growing tissues “outside of the body of which they were once part” [4]. In 1961, the *Guardian* noted how “human embryos ending their existence at London hospitals are providing scientists with the means of making a vaccine against the common cold” [6]. This article detailed how doctors presiding over surgical abortions notified scientists, “who promptly collect the specimens in cold containers, tease out the still-living cells, and dispatch them to the cold research unit at Salisbury” (ibid). Even when the use of tissue from aborted foetuses was outlined in depth, newspapers voiced no criticism. To the *Daily Express*, the use of adult and foetal tissues in biomedical research was “essential and *causes no concern*” [7: Emphasis in original].

These reports are significant, as they undermine claims that research on human tissues invariably met a deep-seated public resistance. They dovetail with the recent findings of historians who have shown that other uses of human tissue did not attract any criticism for much of the twentieth century. Lynn Morgan has detailed how patients in the early twentieth century did not imbue aborted or miscarried foetuses with any significance, but perceived them as waste material. This allowed embryologists to collect them with impunity, and on the rare occasions that researchers asked for specimens their requests were nearly always granted [32]. Susan Lederer, meanwhile, has shown that the high-profile acquisition of human tissue for transplantation and grafting was similarly uncontested. Patients and journalists did not articulate concerns about the retrieval of tissue, and newspapers celebrated the technical aptitude that underpinned these procedures [28].

Instead of a longstanding divide, history reveals a homology between scientific practices and popular representations of research on human tissues. In line with positive attitudes to science and medicine after the Second World War, which Roy Porter [38] calls an era of “swashbuckling optimism”, researchers and journalists both portrayed techniques like tissue culture as a “technical advance of first-rate importance” [5]. In surgical theatres, scientific publications and newspaper reports, excised tissues were comprehensively treated as raw materials of biomedical research. The issues of consent and property we encounter today were not concerns for most of the twentieth century, either for scientists or the journalists who covered their work. Pointing out that informed consent was overlooked in this period is therefore unfair on earlier generations who worked to different standards, in a different climate, and leads us down a blind alley. A more fruitful approach would surely be to explain why these ethical standards have acquired significance in recent decades.

## Calls for Informed Consent in the 1970–1990s

Demands that researchers should seek patient consent before undertaking research on tissues did not emerge thanks to some longstanding public unease, but stemmed from specific sociopolitical changes in the 1970s and 1980s. One major impetus was the pro-life backlash to the liberalisation of American abortion laws in the 1973 *Roe* v *Wade* case. Pro-life campaigners argued that research on foetal tissues was “as outrageous as research on the living fetal being” [39: 67] and should be banned, since it entrenched acceptance of abortion among the medical community and the public. Opponents of research picketed laboratories that were known to use foetal tissue cultures, and successfully evoked an old grave robbing law in Massachusetts to secure an indictment against three scientists who obtained aborted foetuses for research in 1974 [21]. Despite protestations that no ethical guideline had been breached, the assistant District Attorney maintained that “had the researchers asked each woman for permission to perform what amounts to an autopsy on her dead, aborted foetus, there would be no case” [15: 420]. Although the researchers were acquitted, scientists now questioned whether they were secure in obtaining foetal tissues without seeking parental consent.

The National Institutes of Health (NIH) subsequently recommended that doctors and scientists should obtain parental consent before undertaking research on tissues from aborted or miscarried foetuses. This was partly an attempt to forestall another ‘graverobbing’ case, but it also reflected the emphasis on individual autonomy that increasingly characterised American debates on the rights of patients and experimental subjects in the 1970s. Following the outcry over the non-consensual withholding of drugs from African-American men in the notorious ‘Tuskegee syphilis study’, which came to light in 1972 and prompted calls for external oversight of biomedical research, growing numbers of theologians, lawyers and philosophers became public authorities on scientific and medical ethics. In federal committees, new journals and guidelines such as the *Belmont Report*, these ‘bioethicists’ made respecting patient autonomy a guiding principle for all doctors and biomedical researchers.

Perhaps inevitably, certain bioethicists began to question whether patients had a right to control the fate of excised surgical materials and urged medical researchers to adopt informed consent measures to ensure that tissue culture research appeared “legally permissible and morally sensitive” [53], either by approaching patients before operations or distributing consent forms to the resident surgeon. While some researchers dismissed this as a “foolish restriction”, others agreed that securing patient consent would prevent questions about “who has rights in cut hair, nail clippings and cells taken from human bodies” [43]. They viewed consent here as facilitating a form of property transfer: with patients having some legitimate rights over excised tissues, but transferring these to scientists once they granted permission for research.

Tellingly, it was a biologist, and not a patient or bioethicist, who first raised the possibility of patient or family ownership of tissue cultures in a legal setting. The scientist in question was Leonard Hayflick, who was embroiled in a long-running dispute with the NIH over the ownership of WI-38, a foetal tissue culture he had established in the 1960s using tissue obtained from an abortion performed in Sweden. The dispute originated when Hayflick began to sell WI-38 for profit in the 1970s. The NIH argued that it was the rightful owner of WI-38 and should profit from sales as Hayflick had established and maintained the culture using federal funds; while Hayflick countered that he was the rightful owner as he had performed the crucial task of transforming the tissue sample into a viable scientific tool. During 5 years of legal hearings, Hayflick and his lawyers argued that WI-38 might also be considered the property of the Swedish couple whose aborted foetus had originally provided the tissue. As he later wrote [23: 196]

We argued, I believe for the first time, that not only did my former institution and I have a legitimate claim to these cells, but a good case could also be made for title to be vested in the parents of estate of the embryo from which WI-38 was derived.

This complicates a major theme in the ‘social unease’ literature, which portrays the demand for patient consent or ownership as emanating from outside science and medicine, as part of a general public demand to control the fate of excised body parts.

Questions surrounding consent and property did not become linked to a ‘public’ desire to control tissue samples until the mid 1980s, during coverage of the long-running *John Moore* v *Regents of the University of California* case. Here again, views on appropriate ethical safeguards traversed professional boundaries. Some scientists supported Moore’s demand for property rights in his excised spleen cells, while others dismissed it; and this lack of consensus was also evident in law and bioethics. Some bioethicists, including Arthur Caplan, supported new consent protocols but rejected a more lasting property interest in tissues. But others, including Lori Andrews, a *pro bono* member of John Moore’s legal team, called for the adoption of a free-market model where patients owned excised tissues and could sell them to doctors or scientists. Andrews notably endorsed her proposals by claiming that “people have an interest in what happens to their extracorporeal body parts” [1: 32]. Yet the extent to which *Moore* reflected a widespread public desire was questionable. To support her argument that people cared about the fate of tissues, Andrews cited an opinion poll where only 20 % of respondents expressed unease at its use in research.

British scientists and bioethicists did not consider issues of consent and property until after the *Moore* case, partly thanks to the fact that pro-life activists lacked influence in the 1970s and did not target research, and partly thanks to the fact that ‘bioethics’ did not emerge as a recognised term or approach here until the late 1980s [52]. Following *Moore* the majority of British scientists and bioethicists argued that patients should be denied property rights in tissues, and that clinical consent forms should simply be modified to note the possibility of research on excised materials. Justification for this position in Britain, where healthcare is publicly funded and free at the point of access, had distinctly utilitarian overtones. Ethicists and scientists often framed tissue research as a communal endeavour “in which researchers and research participants contribute to research that will contribute to public health” [48: 25]. This argument again drew on projections of ‘public opinion’ for support. In a 1995 report, for example, the recently established Nuffield Council on Bioethics [34: 68] argued that new consent measures were unnecessary since “in the general run of things a person from whom tissue is removed has not the slightest interest in making any claim to it”.

These divergent standpoints suggest that readings of ‘public opinion’ are not universal or self-evident and cannot be dissociated from the ethical standpoint of the individual or group endorsing a specific proposal. What is more, these proposals are historically and socially contingent. The communitarian arguments of the Nuffield Council, for one, reflected the longstanding belief that British patients would support research and contribute to medical progress in exchange for the ‘protection against deprivation, ignorance and disease’ they received from the welfare state [31]. The free-market arguments of Lori Andrews, by contrast, reflected a neo-liberal view of patients as ‘active consumers’ that was more prevalent in American healthcare and bioethics [46]. In a climate where individual autonomy was a dominant value, and where blood had been considered private property since the 1950s, Andrews and others believed it made sense to extend the rationality of the market to the acquisition of human tissues.

With this in mind, we should exercise caution when authors today claim to be speaking for ‘the public’. Accounts that emphasise alientation, exploitation and commodification, including many histories of HeLa, come with their own agendas and miss an important part of the story by not providing insight into how people in specific times and places behave and make sense of their decisions. More empirical studies show that attitudes to tissue research vary according to time and place and, more specifically, to the ontological status of tissue samples and the relationships patients and their families have with biomedical researchers. In Britain, for instance, recent interviews with parents of children undergoing cancer treatment show they view tumour samples as external to their child and as having an intruder status. Many strongly support the use of these tissues in research and see it as a means of reciprocating the free care their children receive: aligning themselves with a particular social world that includes doctors, nurses and researchers.^3^

But these respondents also said their attitudes may change in a different healthcare setting where they paid for their children’s treatment, or if researchers wanted to utilise body parts more strongly linked to notions of personhood, such as hearts or brains.^4^ This reaffirms that we should exercise caution when using select cases as proof of a widespread and general public sentiment. Human tissues today can be used in countless projects, in the arts as well as sciences, and their meanings vary according to the particular significance they hold for specific actors at different times and places: for clinicians, patients, scientists, museum audiences, pro-life activists, the media, bioethicists and so on. Today, as before, views on the appropriate uses of tissue consistently differ *within* and overlap *between* these groups, in line with changing professional and social contexts. Instead of locating tissues in a dichotomy between science and its publics, we would do better to acknowledge the diverse ways in which it binds them together.

## Conclusions

Much of the recent work on the history of tissue culture erects an untenable barrier between material practices and their wider culture: overlooking how the exchange of human materials reflected and helped constitute a broader fabric of social relations. As Catherine Waldby and Robert Mitchell state, the processes of collection, storage and research that human tissues undergo have long been shaped by the attitudes of a variety of groups within and outside of biomedical science [50]. Since the collection and use of human tissue was widely covered by newspapers and other public sources for much of the twentieth century, and since it attracted no criticism, it would appear that the views of these groups did not conflict as much as we have been led to believe.

Accounts that cite the non-consensual acquisition of tissues as evidence of professional malpractice, with HeLa the obvious example, rather miss the point. Uprooting contemporary ethical principles, like getting informed consent for research on tissues, and applying them to periods where they did not exist undermines our appreciation of the past in critical ways. It projects a current view of the world backwards and overlooks how historical actors lived and worked in a different moral climate. The historians Roger Cooter and Claudia Stein [14: 166–167] neatly critique this presentist viewpoint when they argue that “this is not the history that inhabitants of the past understood; it is ours”. Just as importantly, these accounts also distort our understanding of the present. They presume that current ethical principles like informed consent are historically transcendent and, in doing so, neglect the ways in which “moral judgements are made within a conceptual framework which is itself the creation of history” [11: 82].

Yet there is more at stake here than simply dismissing the spectre of ‘Whiggish history’. By portraying scientists and doctors as shadowy figures who regularly contravene popular norms, the ‘social unease’ literature runs the risk of deepening what several ethicists and social scientists identify as a ‘culture of mistrust’ towards science and medicine today. As Onora O’Neill [35: 128] notes, recent controversies surrounding GM crops, stem cell research and the origins of BSE, among others, all point to a “systematic crisis of trust” in scientists and doctors. But O’Neill outlines how there is good evidence that those who seek to remedy this apparent crisis only make it worse, by portraying health professionals as untrustworthy agents who “pursue their own interests rather than those of patients or the public” [35: 3].

By refuting this false dichotomy between science and the public, and portraying tissue cultures as objects that facilitate engagement between professional and popular interests, history might help us foster O’Neill’s [35: 158] goal of a “culture of solidarity” where scientists, patients and the broader public engage in a sustained dialogue on the removal, storage and biomedical uses of tissues. This dialogue is vital to ensuring public trust in an era when human tissues remain central to biomedical research and our ‘knowledge economy’. Today, more than ever, governments, funding bodies, scientists and patients invest great hopes in research that depends on tissues and cells, such as tissue engineering, stem cell research, vaccine development or the construction of large-scale ‘biobanks’. Confidence in these projects, and the vital supply of research materials from patients and volunteers, may falter if we continue to believe that scientists and the public have long been at loggerheads. In rejecting anachronistic moral evaluations, then, historians can do more than ensure scientists and doctors from the past receive a fair hearing, and can introduce much needed balance to contemporary debates on tissue research.

## Footnotes

1 Landecker [27].

2 Ibid.

3 Dixon-Woods et al. [16].

4 Ibid.
